# Population Structure in the Roundtail Chub (*Gila robusta* Complex) of the Gila River Basin as Determined by Microsatellites: Evolutionary and Conservation Implications

**DOI:** 10.1371/journal.pone.0139832

**Published:** 2015-10-16

**Authors:** Thomas E. Dowling, Corey D. Anderson, Paul C. Marsh, Michael S. Rosenberg

**Affiliations:** School of Life Sciences, PO Box 874501, Arizona State University, Tempe, Arizona, United States of America; University of Arkansas, UNITED STATES

## Abstract

Ten microsatellite loci were characterized for 34 locations from roundtail chub (*Gila robusta* complex) to better resolve patterns of genetic variation among local populations in the lower Colorado River basin. This group has had a complex taxonomic history and previous molecular analyses failed to identify species diagnostic molecular markers. Our results supported previous molecular studies based on allozymes and DNA sequences, which found that most genetic variance was explained by differences among local populations. Samples from most localities were so divergent species-level diagnostic markers were not found. Some geographic samples were discordant with current taxonomy due to admixture or misidentification; therefore, additional morphological studies are necessary. Differences in spatial genetic structure were consistent with differences in connectivity of stream habitats, with the typically mainstem species, *G*. *robusta*, exhibiting greater genetic connectedness within the Gila River drainage. No species exhibited strong isolation by distance over the entire stream network, but the two species typically found in headwaters, *G*. *nigra and G*. *intermedia*, exhibited greater than expected genetic similarity between geographically proximate populations, and usually clustered with individuals from the same geographic location and/or sub-basin. These results highlight the significance of microevolutionary processes and importance of maintaining local populations to maximize evolutionary potential for this complex. Augmentation stocking as a conservation management strategy should only occur under extreme circumstances, and potential source populations should be geographically proximate stocks of the same species, especially for the headwater forms.

## Introduction

In deserts of western North America, long periods of aridity have been punctuated by occasional wet interludes [1], leading to fluctuating levels of habitat connectivity within terrestrial and aquatic environments over the last 2–3 million years. Glacial cycles (at approximately 100 kyr intervals) are correlated at middle latitudes with pluvial cycles, causing relatively regular patterns of isolation and connectedness [2]. For some taxa, fluctuating levels of isolation combined with ecological opportunity in arid environments are thought to have resulted in elevated rates of lineage diversification [3–6]. However, in North American freshwater fishes, species richness is lower west of the continental divide, as only about 150 of 750 species reside there [7]. This pattern has been influenced, in part, by tectonic activity and severity of the environment, leading to elevated extinction rates [8]. More recently, human actions have exacerbated this situation, and freshwater and diadromous fishes of North America are declining at an alarming rate [9]. Approximately 39% of described taxa are considered imperiled, representing a 92% increase since 1989. The situation is especially dire as 89% of imperiled taxa listed in 1989 exhibit the same status or worse, indicating that little has been achieved in the past quarter century to improve the status of most endangered fishes.

An interesting case involves the roundtail chub: a complex of closely related species of the cyprinid genus *Gila* (*G*. *robusta*, *G*. *intermedia*, and *G*. *nigra* [the last formerly *“grahami”*]) that are endemic to the Gila River basin in the southwestern United States. Like most other fishes of the region, the geographic distribution of populations has been reduced dramatically by human impacts; numbers are dwindling throughout their ranges, and remaining populations face myriad threats to their persistence [10, 11]. This has resulted in listing *G*. *intermedia* as endangered [12], petitions to similarly list *G*. *nigra and G*. *robusta* [13, 14], and inclusion of one or all species in regional conservation plans [15, 16].

The three species of the *G*. *robusta* complex provided an excellent opportunity for examining the distribution of genetic variation in threatened and endangered species. These species have had a complex and confused taxonomic history, and several detailed studies of variation of meristic and morphological traits have been completed [17–19]. In addition, DeMarais [20] examined genetic variation at 25 presumptive allozyme loci within and among populations of this complex. Analysis of the distribution of genetic variation identified significant differences among locations (*F*_ST_ = 0.410), however, this analysis did not identify significant structure associated with hydrogeography or species. Given observed distributional patterns and levels of genetic variation, DeMarais [20] hypothesized that the form “*grahami*” arose through past introgression between *G*. *intermedia* and *G*. *robusta*.

Minckley and DeMarais [21] summarized available distributional, morphological, and molecular data and examined the taxonomic status of all three species of the complex. Because each morphologically discrete form was consistently collected at the same locations and was always allopatric, they concluded that *G*. *intermedia*, *G*. *robusta*, and “*grahami*” represented three distinct taxonomic species. They also noted that some type specimens of “*grahami*” were actually *G*. *robusta*, invalidating this nomen; the earliest available replacement name was *Gila nigra*. Minckley and DeMarais [21] further discuss origins of *G*. *nigra*, hypothesizing that it may have multiple, independent origins through discrete hybridization events between *G*. *intermedia* and *G*. *robusta*.

Schwemm [22] found similar results to those of DeMarais [20] when he characterized sequence variation of mtDNA and two nuclear loci (introns of S7 and TPI). He found limited divergence among alleles/haplotypes; however, many locations exhibited unique variants, and were frequently monomorphic for these private alleles/haplotypes. Hierarchical analysis failed to associate patterns of sequence variation with species or hydrogeographic connection, and patterns of variation were best explained by fragmentation and independent evolution of local subpopulations.

The present study extends the existing population genetic literature on the *G*. *robusta* complex by providing an analysis of microsatellite DNA loci for the same samples used by Schwemm [22]. Our results demonstrate differences in spatial genetic structure between the mainstem form (*G*. *robusta*) and the headwater forms (*G*. *intermedia and G*. *nigra*) that have important implications for managing local populations associated with different types of desert stream habitats. The patterns we observed are consistent with patterns of gene flow reported for many desert fishes, where genetic connectivity is a function of hydrogeographic connectivity, and often varies between populations occupying different desert stream environments [23]. Our findings have important implications for conservation management the *G*. *robusta* complex and other aquatic organisms that inhabit desert stream environments.

## Materials and Methods

### Ethics Statement

Permission to undertake field work and collect specimens was obtained under permits from the states of Arizona and New Mexico, and the U. S. Fish and Wildlife Service (FWS Federal Fish and Wildlife Service Native Endangered Species Recovery Permit Number TE0-39716-1). Specimens were obtained under Arizona State University Institutional Animal Care and Use Committee (IACUC) approval 05-768R.

### Study organisms

The three species studied here were once common inhabitants of lower Colorado River basin streams and rivers. *Gila robusta* historically was found in moderate size and larger mainstem waters including Bill Williams, Gila, Salt, San Pedro and Verde rivers in the lower Colorado River basin and in Colorado, Gunnison, San Juan, and Green rivers in the upper basin where it occupied the largest, deepest pools and attained more than 45 cm total length. Smaller individuals inhabited smaller habitats, as with many western fishes [24], where they prefer shaded, deeper pools with cover such as undercut banks, boulders, or debris. Best described as a creek fish, *Gila intermedia* is a Gila River basin endemic that occupies well-developed pools with abundant cover in small to middle-size headwater creeks [25]; it is most common in marshy areas and ciénegas. *Gila intermedia* and *G*. *robusta* have never been taken syntopically despite spatial proximity of some populations. *Gila nigra*, also a Gila River basin endemic, inhabits smaller, first-order to middle reaches of medium sized streams, where it is strongly associated with cover such as undercut banks, boulders, and debris. It does not occur in ciénegas and does not co-occur with either of its congeners.

### Sampling and DNA extraction

**Fig 1:**
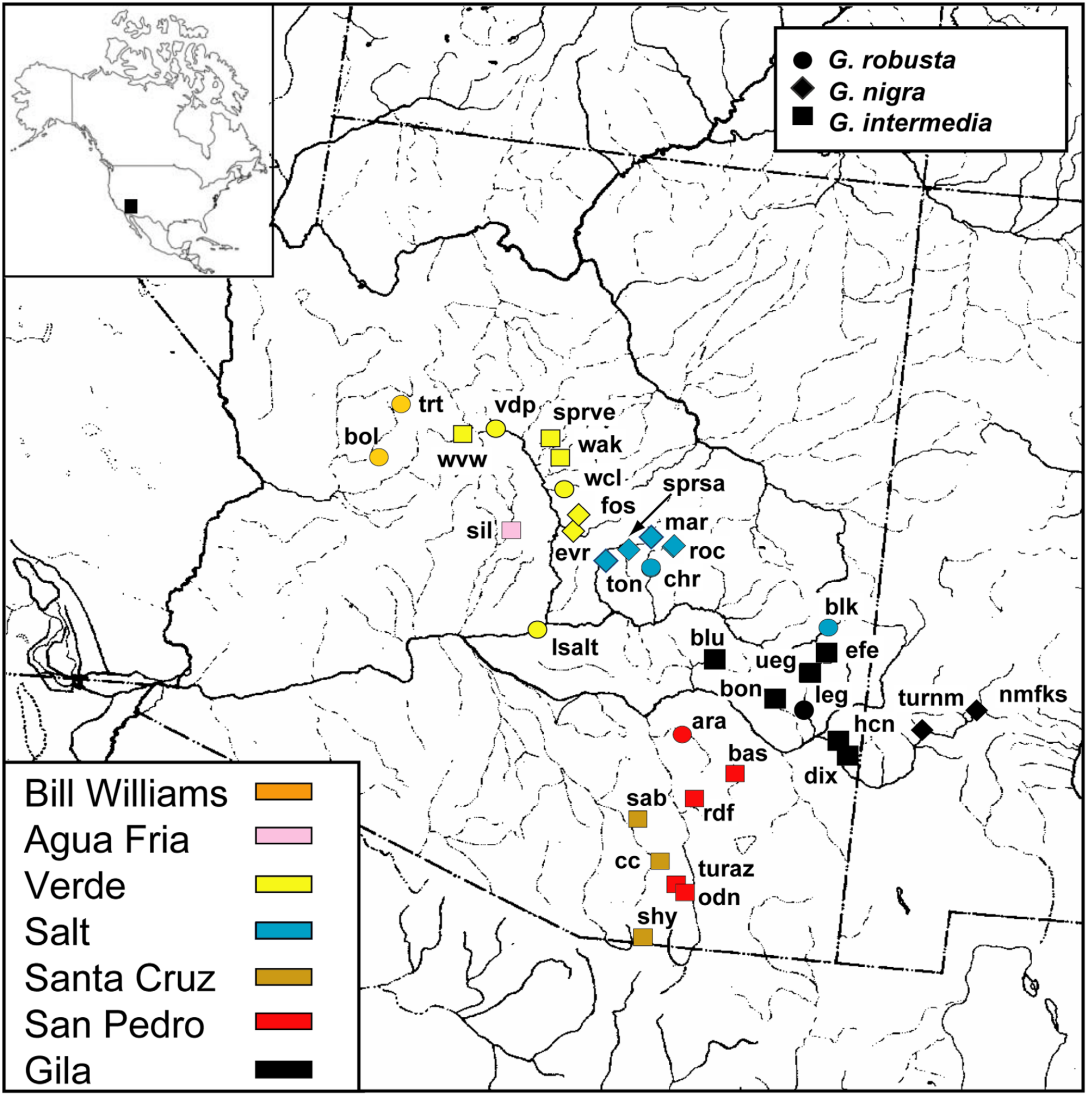
Locality map for samples characterized in the study of the *Gila robusta* complex from Arizona and New Mexico. Approximate locations are identified by symbols with shape and color indicating species and drainage unit, respectively (see legends for detailed information). Locality data are provided in Table 1. Reprinted from the Fish Division drainage map, University of Michigan Museum of Zoology, under a CC BY license, with permission from University of Michigan Museum of Zoology, original copyright 1972.

Sampling of Gila and Bill Williams river drainages encompassed 34 sites in seven sub-basins in Arizona and New Mexico (Bill Williams, Agua Fria, Verde, Salt, Santa Cruz, San Pedro, and Gila River mainstem–Fig 1), representing most known extant and some extirpated populations of these taxa. Minckley and DeMarais [21] summarized information meristic, morphometric and pigmentation characters of samples from locations examined here and identified them to species; therefore, we follow their taxonomic designations.

Efforts were made to sample up to 25–30 individuals/locality; however, the rarity of these species sometimes made it difficult to achieve this goal. Fourteen to 30 individuals were collected from each locality (Table 1). Some of these locations were represented by frozen tissues from whole specimens collected in the 1980s for an allozyme study by DeMarais [20]. Specimens obtained for DNA studies (Schwemm [22] and here) were captured using standard fisheries methods (i.e., electrofishing, seining, or trapping). Tissues were obtained by removing a piece of right pectoral fin (< 3 mm square) from larger individuals with ethanol sanitized surgical scissors; after which fish were immediately released unharmed. This process was fast (a few seconds), required minimal handling, and caused no harm to the fish so no anesthesia was applied. For larvae/young-of-year, specimens were euthanized in 500 mg/L MS-222. All material was transferred immediately after acquisition to 95% ethanol for storage. Genomic DNA was extracted from tissues by standard proteinase K/phenol/chloroform protocol as modified by Tibbets and Dowling [26].

**Table 1:** Locality data and groupings of samples for hierarchical analyses of samples from the *Gila robusta* complex, Arizona-New Mexico. All sub-basins are within the Gila River basin except for the Bill Williams River, which is a direct tributary to the lower Colorado River. Taxonomic identity of sampled individuals follows Minckley and DeMarais [21]. Coordinates are provided in UTM, elevation is in meters, and the column “N” provides number of individuals analyzed, with the superscript identifying the original source of material.

Location	Acronym	Coordinates (UTM)	Elevation	Species	N
***Gila River basin***					
***Agua Fria River basin***					
Silver Creek, Yavapai Co., AZ	SIL	12S 414762E 3793564N	1301m	*intermedia*	29^S^
***Gila River sub-basin***					
Blue River, Gila Co., AZ	BLU	12S 566895E 3708078N	1261m	*intermedia*	19^S^
Bonita Creek, Graham Co., AZ	BON	12S 637253E 3647293N	1077m	*intermedia*	20^D^
Dix Creek, Greenlee Co., AZ	DIX	12S 671832E 3675085N	1200m	*intermedia*	22*
East Fork Eagle Creek, Greenlee Co., AZ	EFE	12S 643185E 3707050N	1716m	*intermedia*	20^D^
Harden-Cienega Creek, Greenlee Co., AZ	HCN	12S 673853E 3674850N	1210m	*intermedia*	22^S^
Eagle Creek—lower, Greenlee Co., AZ	LEG	12S 648752E 3651019N	1020m	*robusta*	20^S^
East, Middle and West Forks Gila River, Catron Co., NM	NMFKS	12S 758619E 3677745N	1713m	*nigra*	19^S^
Turkey Creek, Grant Co., NM	TURNM	12S 734293E 3662829N	1462m	*nigra*	18^S^
Eagle Creek—upper, Greenlee Co., AZ	UEG	12S 641212E 3704936N	1653m	*intermedia*	18^S^
***Salt River sub-basin***					
Black River, Greenlee Co., AZ	BLK	12S 639590E 3724720N	2003m	*robusta*	21^S^ ^,^ *
Cherry Creek, Gila Co., AZ	CHR	12S 515036E 3740368N	917m	*robusta*	21^D^
Marsh Creek, Gila Co., AZ	MAR	12S 497689E 3780336N	1519m	*nigra*	27^S^
***Salt River sub-basin***					
Rock Creek, Gila Co., AZ	ROC	12S 489588E 3759443N	1586m	*nigra*	20*
Spring Creek, Gila Co., AZ	SPRSA	12S 495954E 3765606N	1428m	*nigra*	20^S^
Tonto Creek, Gila Co., AZ	TON	12S 491190E 3786183N	1229m	*nigra*	16^S^
***Santa Cruz River sub-basin***					
Cienega Creek, Pima Co., AZ	CC	12S 540260E 3524841N	1280m	*intermedia*	20
Sabino Canyon, Pima Co., AZ	SAB	12S 519663E 3577319N	940m	*intermedia*	14
Sheehy Spring, Santa Cruz Co., AZ	SHY	12S 540028E 3470448N	1433m	*intermedia*	25
***San Pedro River sub-basin***					
Aravaipa Creek, Pinal Co., AZ	ARA	12S 551774E 3640104N	922m	*robusta*	24
Bass Canyon, Cochise Co., AZ	BAS	12S 571383E 3579679N	1234m	*intermedia*	20
O’Donnell Canyon, Santa Cruz Co., AZ	ODN	12S 544969E 3491596N	1505m	*intermedia*	20
Redfield Canyon, Pima Co., AZ	RDF	12S 562895E 3588722N	1111m	*intermedia*	20
Turkey Creek, Santa Cruz Co., AZ	TURAZ	12S 546154E 3489828N	1516m	*intermedia*	18
***Verde River subbasin***					
East Verde River, Gila Co., AZ	EVR	12S 465504E 3794077N	1355m	*nigra*	20
Fossil Creek, Yavapai Co, AZ	FOS	12S 447363E 3861552N	1328m	*nigra*	26
Canal downstream from confluence Salt and Verde rivers, Maricopa Co., AZ	LSALT	12S 438441E 3712009N	404m	*robusta*	29
Spring Creek, Yavapai Co, AZ	SPRVE	12S 415889E 3853475N	1170m	*intermedia*	20
Verde River, Perkinsville, Yavapai Co, AZ	VDP	12S 391039E 3862667N	1163m	*robusta*	20
Walker Creek, Yavapai Co, AZ	WAK	12S 435967E 3833635N	1227m	*intermedia*	24
West Clear Creek, Yavapai Co, AZ	WCL	12S 436195E 3822226N	1102m	*robusta*	29
Williamson Valley Wash, Yavapai Co, AZ	WVW	12S 364924E 3822226N	1102m	*intermedia*	20
***Bill Williams River basin***					
Boulder Creek, Yavapai Co., AZ	BOL	12S 302702E 3834064N	1156m	*robusta*	30
Trout Creek, Mohave Co., AZ	TRT	12S 275979E 3875303N	1145m	*robusta*	30

^D^ = DeMarais,

^S^ = Schwemm,

* = this study

### Microsatellite loci

Primers for ten microsatellite loci used here were derived from several sources. Six loci (*36*, *222*, *223*, *225*, *227*, *300*) were developed by Keeler-Foster et al. [27] from a *G*. *elegans* library. One locus (*G294*) was developed by Meredith and May [28] from a *G*. *bicolor obesa* library. The remaining three loci, *C2* (repeat unit, (GACA)_4_GTCA(GACA)_3_(GATA)_3_; primers 5'-GACAAAGCGGTAGACAAAACCA-3' and 5'-AATCTGAACTGGCTAACCTT-3'), *D17* (repeat unit, (GT)_13_; primers 5'-TGGGCAGGAAAAGAGAAACT-3' and 5'-ATAAAGAGACGGTAAAGAACTC-3'), and *D42* (repeat unit, (TCTA)_5_, primers 5'-TTGCCTGTATAGGGTTGA-3' and 5'-GTTGCTCATTGTTAGTTTGT-3'), were obtained from a library generated from *G*. *robusta* using enrichment methods provided by Glenn and Schable [29]. Amplifications used GoTaq (Promega) and the buffer supplied, dNTPs (200 mM final concentration of each dNTP), and IRD labeled primers (0.5 μM final concentration). Reactions were started with a long denature step (95°C, 5 min) followed by a series of touchdown steps where annealing temperature was decreased 1 C each cycle (94°C, 30 sec; 65–50°C, 30 sec; 72°C, 30 sec) to a final temp of 50°C. These same steps were repeated for additional cycles until 25 or 30 total cycles were completed, and the run finished with a long extension step (72°C, 7 min). Products were separated by electrophoresis through 6.5% denaturing gels (KB^Plus^, LI-COR Biotechnology) for 90–105 mins at 40 W with a minimum of four ladder lanes (50 bp—350 bp size standard, LI-COR Biotechnology) included on each gel. Fragments were visualized on a LI-COR 4300 DNA Analysis system and analyzed using SAGA GT (version 3.3, LI-COR Biotechnology).

### Statistical analyses

Deviations from Hardy-Weinberg equilibrium (*F*_IS_) and multilocus equilibrium were examined using FSTAT version 2.9.3.2 [30]. Significance level (0.05) for single and multilocus tests was adjusted using the B-Y correction [31]; adjusted critical values of 0.00797 and 0.00653, respectively). The unbiased estimate of gene diversity [32] and allelic richness (*A*_R_—corrected for sample size by rarefaction) were calculated using FSTAT and HP-Rare [33], respectively. Basic statistical analyses (e.g., ANOVA, Kruskal-Wallis) were performed using PASW Statistics (formerly SPSS), release 18.

To examine distribution of variation among sample populations we also used FSTAT to generate Weir and Cockerham [34] *F*-statistics. Significance of values for *f* (≈ *F*_IS_), Θ (≈ *F*_ST_), and *F* (≈ *F*_IT_) were obtained by jackknifing (over individuals and all loci) and bootstrapping (loci only). Comparison of the levels of Θ among the three species was obtained by bootstrapping across samples (2500 permutations) using the comparison among groups of samples function in FSTAT. *F*_ST_ was further partitioned by species or drainage using AMOVA with Arlequin version 3.11 [35]. Sample populations were clustered by neighbor-joining using POPTREE2, using corrected *F*_ST_ as the estimate of genetic distance with confidence of nodes assessed by bootstrapping (1000 replicates) [36].

Stream distance were estimated from stream data from the Digital Chart of the World [37] using the network extension in the GIS software ARC/INFO. To test for isolation by (stream) distance, PASSaGE 2 [38] was used to perform Mantel tests and build Mantel correlograms [39–41]. The standardized Mantel statistic (*r*_M_) was used to measure the correlation between pairwise *F*_ST_ and stream distance over all sample populations. Mantel correlograms were constructed with five distance classes with an approximately equal number of pairs in each class. For each distance class, sites belonging to the same distance class received a value of 1 and the other pairs received value of 0 and design matrices were compared to a resemblance matrix based on pairwise *F*_ST_. In this context, the standardized Mantel coefficient (*r*_M_) was used as a measure of spatial autocorrelation for distance data, and can be used in the same way in a correlogram. For both the Mantel test and Mantel correlograms, the statistical significance of the standardized Mantel statistic (*r*_M_) was tested by randomly permuting the rows and columns of one matrix in tandem (= 9999 permutations) and then counting the number of cases that yielded a Mantel coefficient greater than or equal to the observed value.

We also used Bayesian Assignment Tests to determine whether individuals (or groups of individuals) could be sorted into discrete gene pools. Assignment of individuals to gene pools was generated using STRUCTURE version 2.2 [42, 43] and assignment of groups of individuals to demes was examined using BAPS 5.1 [44]. For BAPS 5.1 analyses, separate runs were completed for each species, treating each sample location as an "informed prior." We then combined all sample locations (over all species) and repeated the analysis. For all runs, we entered a vector of replicate *K* values (10 replicates per *K*, from *K* = 2 to *K* = *n*, where *n* is the number of sample populations); BAPS 5.1 reports the set of estimates with the “best” partition and probability associated with different *a priori* assumptions.

For STRUCTURE, the default assumption (admixture among samples, correlated allele frequencies across loci) was employed. For each *a priori* assumed number of populations (*K)*, 10 independent runs of 110,000 replicates each (burn-in = 10,000) were performed. Optimal number of groups (*K*) was determined using the method of Evanno et al. [45] as implemented by the web-based program STRUCTURE HARVESTER [46].The distribution of Q values across runs for each *K* was summarized using CLUMPP [47] and the statistic *H’* calculated to provide assessment of similarity across replicates; results were visualized using DISTRUCT [48].

## Results

### Variation within populations

Genetic variation in *Gila* was characterized using 744 individuals from 34 locations and 10 microsatellite loci. Genotypes for each individual are provided in S1 Table. Most samples had complete data, with an average amplification failure rate of 5.0 individuals/locus or 0.5% of all samples. Locus *300* had the highest failure rate where 15 individuals (or 2% of all individuals) failed to amplify. Failed amplifications were scattered across populations, reducing concerns over potential impact of null alleles.

Average allelic richness per locus was variable across loci (S2 Table), ranging from 1.3 to 7.4 (for loci *C2* and *227*, respectively). Average allelic richness per sample ranged from 1.7 (SAB) to 8.9 (NMFKS), with the majority of lower values reported for *G*. *intermedia* and *G*. *nigra* (Table 2). Populations of *G*. *robusta* exhibited higher levels of variation (*A*_R_ = 6.0) than those of *G*. *intermedia* and *G*. *nigra* (*A*_R_ of 4.7 and 5.0, respectively), with these values significantly different among species (Kruskal-Wallis, *P* = 0.006).

**Table 2:** Population genetic statistics for each sample from the *Gila robusta* complex, Arizona-New Mexico. “Species” follows designations in Minckley and DeMarais [21], “N” is sample size, “A_R_” is allelic richness averaged across loci, “#M” is the number of monomorphic loci, and “HWE” provides the number of significant deficiencies/excesses of heterozygotes per locus for each sample.

Species	Location	Drainage	Acronym	N	A_R_	#M	HWE
*intermedia*	Silver	Agua Fria	SIL	29	2.9	1	1/0
*intermedia*	Blue	Gila	BLU	19	3.5	2	0/0
*intermedia*	Bonita	Gila	BON	20	6.0	1	0/0
*intermedia*	Dix	Gila	DIX	22	4.2	0	1/1
*intermedia*	E Fork Eagle	Gila	EFE	20	7.2	0	0/0
*intermedia*	Harden-Cienaga	Gila	HCN	22	4.1	1	1/0
*intermedia*	Upper Eagle	Gila	UEG	18	6.8	0	0/0
*intermedia*	Bass	San Pedro	BAS	20	5.0	1	0/0
*intermedia*	O'Donell	San Pedro	ODN	20	6.2	1	0/0
*intermedia*	Redfield	San Pedro	RDF	20	4.3	1	0/0
*intermedia*	Turkey, AZ	San Pedro	TURAZ	20	5.5	0	0/0
*intermedia*	Cienaga	Santa Cruz	CC	20	2.1	5	0/0
*intermedia*	Sabino	Santa Cruz	SAB	14	1.7	5	0/0
*intermedia*	Sheehy	Santa Cruz	SHY	25	2.7	0	0/0
*intermedia*	Spring	Verde	SPRVE	20	7.1	1	0/0
*intermedia*	Walker	Verde	WAK	24	3.5	2	2/0
*intermedia*	Williamson Valley	Verde	WVW	20	6.4	1	2/0
*nigra*	Gila Forks, NM	Gila	NMFKS	19	8.9	0	0/0
*nigra*	Turkey, NM	Gila	TURNM	18	3.6	1	0/2
*nigra*	Marsh	Salt	MAR	27	5.8	0	1/0
*nigra*	Rock	Salt	ROC	20	5.4	0	0/1
*nigra*	Spring	Salt	SPRSA	20	6.4	0	0/0
*nigra*	Tonto	Salt	TON	20	6.5	1	1/0
*nigra*	East Verde	Verde	EVR	20	1.8	5	0/0
*nigra*	Fossil Spring	Verde	FOS	26	1.8	4	0/0
*robusta*	Boulder	Bill Williams	BOL	30	2.5	3	0/0
*robusta*	Trout	Bill Williams	TRT	30	4.0	1	0/0
*robusta*	Aravaipa	Gila	ARA	25	6.8	1	0/0
*robusta*	Lower Eagle	Gila	LEG	19	7.1	1	0/0
*robusta*	Black	Salt	BLK	18	8.3	1	0/0
*robusta*	Cherry	Salt	CHR	21	5.7	0	0/0
*robusta*	Lower Salt	Verde	LSALT	29	6.4	1	0/0
*robusta*	Verde, Perkinsville	Verde	VDP	20	6.7	1	0/0
*robusta*	West Clear	Verde	WCL	29	6.7	1	0/0

Average gene diversity per locus ranged from 0.073 to 0.752 (for loci *C2* and *300*, respectively) while average gene diversity per sample ranged from 0.221 to 0.754 (FOS and UEG, respectively) (S3 Table). Fit to Hardy-Weinberg expectations (as indicated by variation in average *F*_IS_ values across loci and populations) did not vary significantly among species as indicated by variation in average *F*_IS_ values (across loci and populations) for *G*. *robusta*, *G*. *nigra*, and *G*. *intermedia* (*F*_IS_ = -0.008, -0.025, and 0.055, respectively; Kruskal-Wallis, *P* = 0.077). Of 340 individual tests conducted (10 loci, 34 locations), 13 showed deviations from Hardy-Weinberg equilibrium after B-Y correction, with more significant tests identifying heterozygote deficiency than excess (9 and 4, respectively, Table 2). Given the rarity of deviations (< 4% of comparisons) and their scatter across loci (seven loci exhibit deviations), impact from null alleles would be minimal, so all samples and loci were included in remaining analyses.

Remaining deviant samples exhibited significant heterozygote deficiencies. Most locations exhibited only a slight deficiency of hetezygotes, while deviations for *G*. *intermedia* from SAB and WAK were larger (overall *F*_IS_ = 0.268, *P* = 0.0096 and overall *F*_IS_ = 0.140, *P* = 0.0015, respectively). Samples from these locations yielded smaller numbers of alleles (*A*_R_ = 1.7 and 3.5, respectively). At SAB, five loci were monomorphic, while four of five polymorphic loci exhibited a deficiency of heterozygotes that was not statistically significant.

All pairs of polymorphic loci were tested for genotypic linkage disequilibrium within each population, with 21 of 1184 pairwise tests (1.8%) significant after B-Y correction, with nearly half of the significant tests coming from two sample populations: ROC and TURNM (4 and 6 significant pairs, respectively). TURNM was unusual in that eight of the nine polymorphic loci exhibited an excess of heterozygotes, with two of those values significant (overall *F*_IS_ = -0.274, *P* < 0.0001), potentially indicating close relatedness among these individuals. Locus pairs exhibiting significant disequilibrium were not consistent from sample population to sample population, indicating that loci are assorting independently.

### Variation among populations

Partitioning of genetic variation into within and among population components identified significant population structure. Jackknife estimates of total genetic variation (*F* ≈ *F*_IT_) for each locus ranged from 0.211–0.407 (loci *222* and *36*, respectively), with a jackknife average *F* across loci of 0.293 (95% confidence interval 0.249–0.342). The within population component (*f* ≈ *F*_IS_) was small and not significantly different from 0 (range -0.083 [locus *C2*] to 0.075 [locus *36*]), consistent with Hardy-Weinberg equilibrium results discussed above (jackknife average *f* = 0.02, 95% confidence interval -0.01 to 0.048). Therefore, the majority of variation was partitioned among populations: Θ (≈ *F*_ST_) ranged from 0.227 (locus *300*) to 0.384 (locus *C2*) with a significant jackknife average of 0.278 (95% confidence interval 0.249–0.314).

To further examine the role of historical factors and geography, among population variation (*F*_ST_) was partitioned by either taxonomy (three species) or river drainage (seven drainages, Fig 1) to see how these factors explain the distribution of genetic variation (calculated as weighted average across loci). When taxonomy was used to define partitions, the majority of the variation was found among local population within species (*F*_SC_ = 0.271) instead of among species (*F*_CT_ = 0.016). A similar result was obtained when samples were partitioned by drainage, with considerably more variation attributable to samples within drainages (*F*_SC_ = 0.245) than among drainages (*F*_CT_ = 0.052).

**Fig 2:**
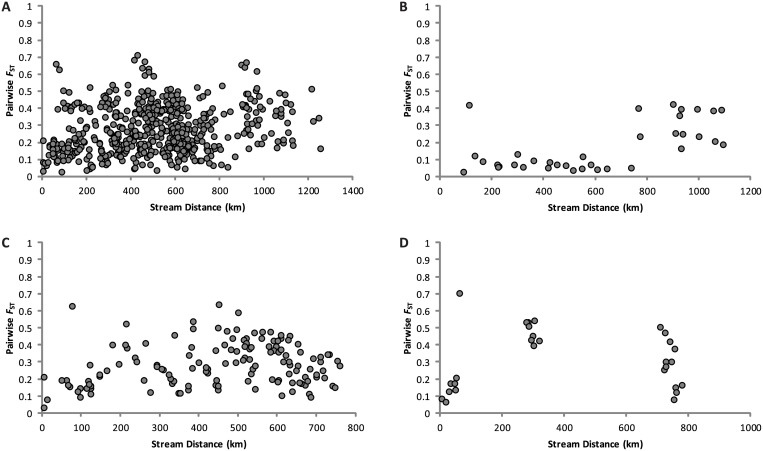
Scatter plots of stream distance against pairwise *F*_ST_ (= *θ*) for samples of the *Gila robusta* complex from Arizona and New Mexico. (A) all species, (B) *Gila robusta*, (C) *Gila intermedia*, (D) *Gila nigra*.

**Fig 3:**
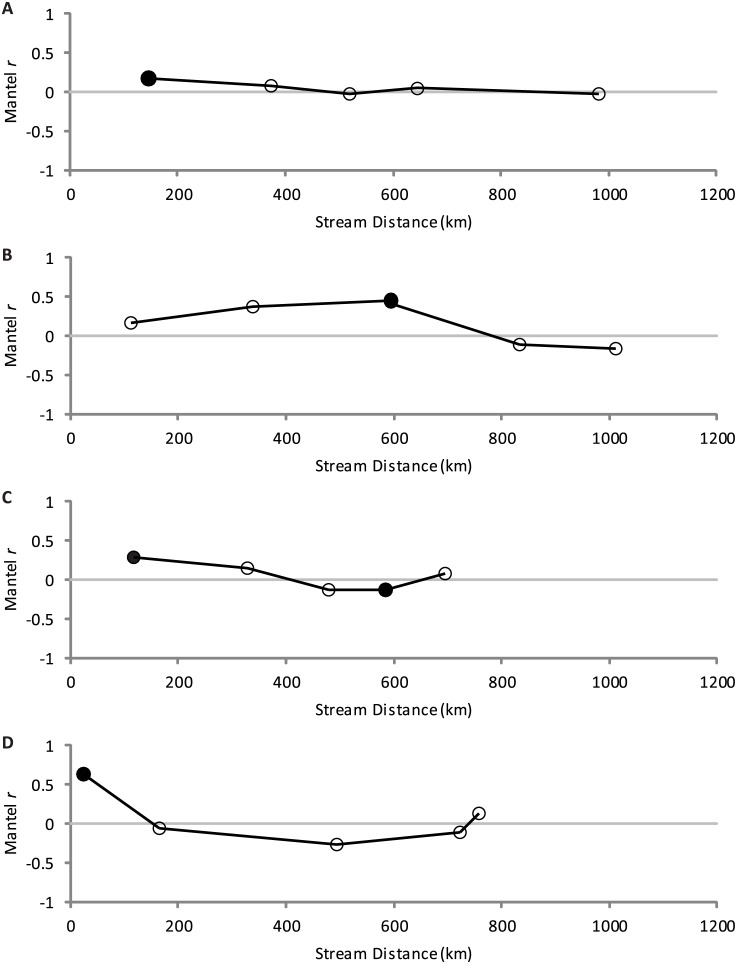
Mantel correlograms for samples of the *Gila robusta* complex from Arizona and New Mexico. (A) all species, (B) *G*. *robusta* (C) *G*. *intermedia*, and (D) *G*. *nigra*. Distance classes with statistically significant (*α* = 0.05) standardized Mantel coefficients are indicated by a filled circle.

When all sample populations from the *Gila* robusta complex were pooled, the spatial correlation between pairwise *F*_ST_ and stream distance was weak and not statistically significant (*r*_M_ = 0.165, *P* = 0.109; Fig 2A). However, when sample pairs were binned into distance classes (Fig 3A), the Mantel correlogram indicated statistically significant standardized Mantel coefficient in the first distance class (*r*_M_ = 0.174; *P* = 0.007).

**Fig 4:**
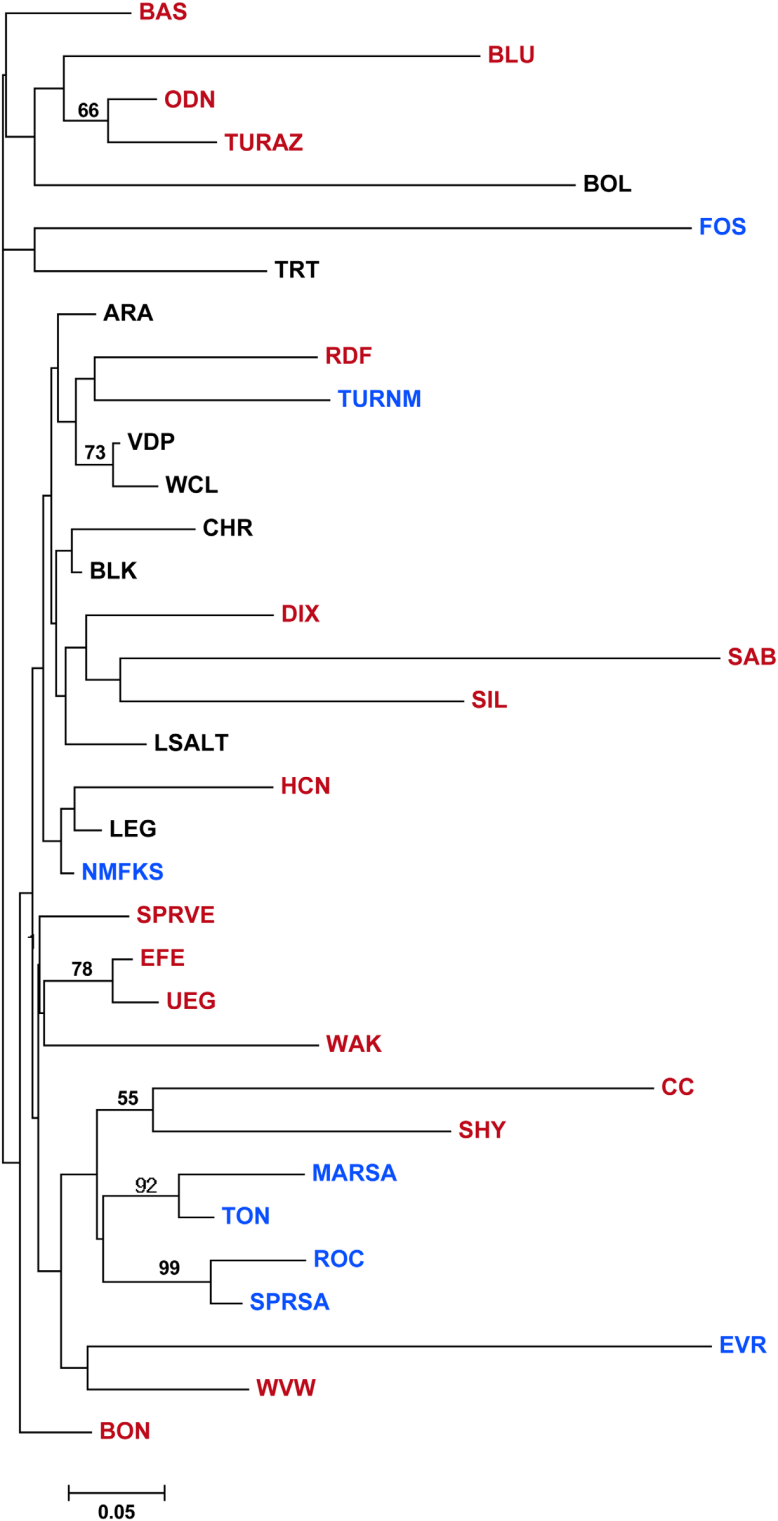
Neighbor-joining network for sample locations of the *Gila robusta* complex constructed using pairwise estimates of *F*_ST_. Location acronyms are provided in Table 1. Red, blue, and black labels and symbols identify samples from *G*. *intermedia*, *G*. *nigra*, and *G*. *robusta*, respectively. Numbers on branches reflect the proportion of 1000 bootstrap replicates in which the defined node was found.

Analysis of population structure independently for each species provides a different picture. Estimates of *F*_ST_ for *G*. *robusta*, *G*. *nigra*, and *G*. *intermedia* were comparable, and not significantly different among species (*F*_ST_ = 0.191, 0.338, and 0.287, respectively; *P* = 0.263). However, when samples from the Bill Williams River drainage (BOL, TRT) were excluded, the average for *G*. *robusta* dropped dramatically (*F*_ST_ = 0.071) and there were significant differences among the three species (*P* = 0.009). This is reflected in the neighbor joining network of pairwise *F*_ST_ values (Fig 4), where many samples of *G*. *nigra* and *G*. *intermedia* exhibited long terminal branches while samples of *G*. *robusta* (except for those from the Bill Williams drainage) were shorter. Most nodes were not supported by bootstrap analysis with the exception of some pairs of samples in relatively close proximity.

For *G*. *robusta*, pairwise *F*_ST_ ranged from 0.03–0.42. When pairwise *F*_ST_ was plotted against stream distance, there were three main clusters of points and one outlier in the scatter diagram (Fig 2B). The main cluster of points corresponded to comparisons between sample locations within the Gila River drainage. The remaining two clusters of points (766 to 1094 km) corresponded to comparisons between Bill Williams River samples (i.e., BOL and TRT) and Gila River samples. The outlier point was the pairwise estimate for BOL and TRT. Over all sample locations of *G*. *robusta*, we found a moderate correlation (*r*_M_ = 0.61) between pairwise *F*_ST_ and stream distance that was statistically significant (*P* = 0.027). When BOL and TRT (from the Bill Williams River drainage) were excluded, the correlation became negative and was not statistically significant (*r*_M_ = -0.24, *P* = 0.237). Differences in connectivity between major drainages are also supported by the Mantel correlogram (Fig 3b), where the standardized Mantel coefficient decreased precipitously for comparisons between sample populations in the Gila and Bill Williams drainages.

For *G*. *intermedia*, pairwise *F*_ST_ ranged from 0.034–0.638. Over all sample locations, the correlation between pairwise *F*_ST_ and stream distance (*r*_M_ = 0.20) was weak and not statistically significant. The Mantel correlogram (Fig 3C) displayed a decreasing trend in the value of *r*_M_ with increasing stream distance. For *G*. *nigra*, pairwise *F*_ST_ ranged from 0.066–0.702. Although sparse, scatterplot shape (Fig 2D) resembled the pattern for *G*. *intermedia* (Fig 2C). The correlation between pairwise *F*_ST_ and stream distance was weak (*r*_M_ = 0.20) over all sample populations and not statistically significant. In the Mantel correlogram, the standardized Mantel statistic was significant only for the first distance class (Fig 3D; *r*_M_ = 0.680, *P* = 0.001).

### Assignment testing

**Fig 5:**
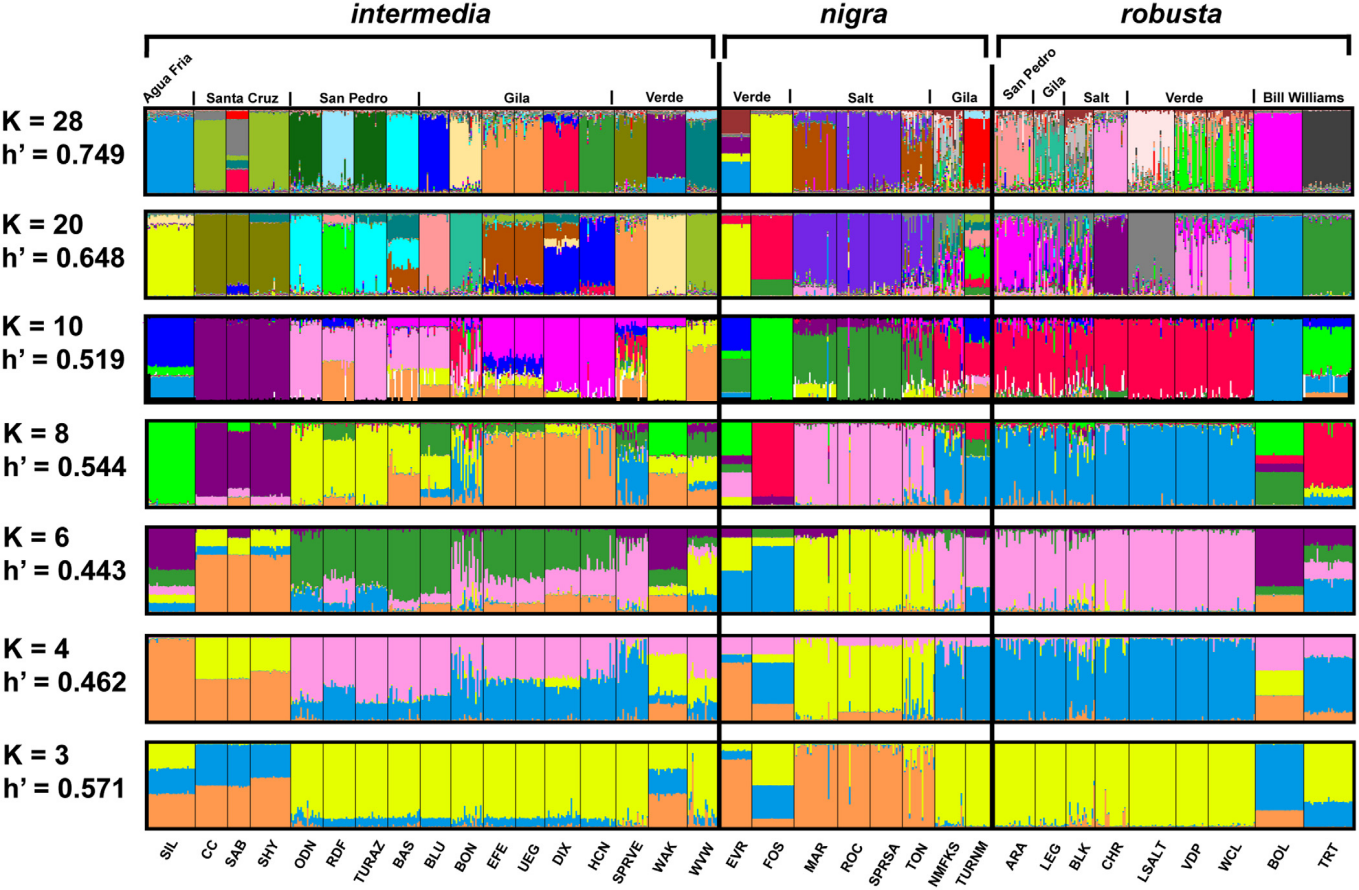
Assignment probability plots for all sample locations of the *Gila robusta* complex, Arizona-New Mexico, for selected values of *K*. “*K*” represents the number of informed priors for that specific group of replicates and “H΄” is the statistic that measures consistency across replicate runs.

BAPS and STRUCTURE were used to estimate the number of groups encompassed by the 34 samples. BAPS determined that *K* = 28 with each identified group represented by single samples except for two, one containing samples EFE and UEG from *G*. *intermedia* and the other comprised of most *G*. *robusta* samples (ARA, BLK, LEG, WCL, and VDP) and NMFKS from *G*. *nigra*. STRUCTURE was used to characterize assignment probability for all *K* from 2–34. There was inconsistency across replicates for more divergent samples (e.g., BOL and TRT), as indicated by their consistent assignment to different groups for each value of *K* and reduced *h’* values for these replicates (Fig 5). The method of Evanno et al. [45] indicated *K* = 20 (Δ*K* = 16.0), and ln likelihood values also reached a plateau at *K* = 20, supporting that conclusion [42].

Evaluation of assignment probability plots from STRUCTURE is difficult due to variation among replicates and the large number of distinct samples. Analyses from *K* = 20 and *K* = 28 (as predicted by STRUCTURE and BAPS, respectively, Fig 5) yielded similar results, with an increase in number of samples that were distinct for the latter *K*. Even at higher *K* certain sets of geographically proximate locations are consistently grouped together: *G*. *robusta* from the Verde River (WCL and VDP); *G*. *nigra* from Tonto Creek (ROC and SPRSA); and three separate groups of *G*. *intermedia* samples (EFE-UEG from Eagle Creek, ODN-TURAZ from the San Pedro River, and CC-SHY from the Santa Cruz River).

While it is difficult to obtain much information from examination of assignment plots for each *K* (Fig 5), several samples are notable. Individuals from BLK (*G*. *robusta*, Salt River), BON and SPRVE (*G*. *intermedia*, Gila and Verde rivers, respectively), and NMFKS and TON (*G*. *nigra*, Tonto Creek drainage) are routinely difficult to assign to specific groups, especially at low values of *K* (≤ 10). In addition, individuals from three of these locations (BON, SPRVE, TON) exhibit signs of admixture at lower levels of *K* (≤ 10) as there is considerable probability of assignment to a group that includes most samples of *G*. *robusta*. Similar perspective of two sites assigned to *G*. *nigra* (NMFKS, TURNM) indicates that individuals from these locations may actually belong to *G*. *robusta*.

## Discussion

Results of the present study were consistent with previous molecular genetic studies of the *G*. *robusta* complex [20, 22]. Most of the genetic variation was attributable to differences among local populations within species, with minimal differentiation due to the presence of multiple drainages or species in the analysis. However, our results provided new insight into spatial genetic structure among local populations associated with different stream habitats, with evidence of widespread gene flow among local populations of the mainstem form within the Gila River basin, as well as evidence of more recent, maybe even ongoing, gene flow between proximate populations than distant populations within each headwater form.

Characterization of microsatellite variation for 10 loci failed to group samples by recognized species, a result consistent with the allozyme study of DeMarais [20] and Schwemm’s [22] characterization of mtDNA and introns from two single copy nuclear genes. Levels of divergence among populations in this complex were high, and clustering of pairwise *F*_ST_s yields a topology with short internodal and long terminal branches, with limited support for grouping of pairs of most populations, let alone those in the same taxonomic group (Fig 4). High divergence also affects Bayesian group assignment, with assignment of sites to a particular group inconsistent across replicates, yielding a distinctive stacked bar pattern for divergent samples, especially at lower values of *K* (Fig 5). These studies illustrate the importance of local isolation in the evolution of this complex, producing a large number of diagnosably distinct, local populations (*K* = 20 and 28 for STRUCTURE and BAPS, respectively). It is important to emphasize here that these patterns are probably not an artifact of Bayesian methods [49] as indicated by high *F*_ST_ values among populations; nor are they an artifact of microsatellite markers, as Schwemm [22] also noted that many local populations are distinct enough to be diagnosable with unique mtDNA haplotypes and/or nuclear alleles.

Despite the high level of divergence among populations for mtDNA and nuclear sequence data and microsatellites, diagnostic markers were not identified for species. There are a few potential explanations for our inability to identify diagnostic molecular markers. When genetic divergence among subpopulations is large, it is possible that differences among individual subpopulations can obscure differences at deeper hierarchical levels (e.g., species, drainages), reducing the effectiveness of such markers for identifying these higher categories. For example, Hedrick [50] noted that hypervariable markers like microsatellites are less effective at estimating *F*_ST_ due to high levels of variation within populations. Extending this logic further, high levels of divergence among local subpopulations would further reduce the amount of variation available to discriminate among higher order groups (e.g., species). Resolving hierarchy may require a substantially larger data set, with additional markers capable of resolving deeper evolutionary events.

The observed pattern could also reflect how different evolutionary forces have shaped this complex. Genetic structure within the *Gila robusta* complex may represent insularization of a historically panmictic population due to natural and artificial habitat fragmentation, with observed patterns of morphological variation due to convergent selection for a common habitat-based morphotype (e.g., ciénegas, headwater reaches), analogous to convergent selection observed in sticklebacks [51]. Assessment of this hypothesis would require identification of specific genes that are the unit of selection for different habitats as well as additional morphological analyses.

Past introgressive hybridization could also be partly responsible for the observed pattern. These species have never been reported to be sympatric naturally despite occurring in close proximity (e.g., Eagle Creek) [52]. DeMarais [20] and Minckley and DeMarais [21] hypothesized that *Gila nigra* was a taxon of hybrid origin, resulting from introgression between *G*. *intermedia* and *G*. *robusta*. During dry periods, *G*. *intermedia* and *G*. *robusta* are expected to be geographically isolated in headwater and mainstem reaches, respectively; however, during wetter times these species could co-occur and interbreed, producing local hybrid swarms. As streams again became desiccated, these hybrid populations would become isolated in headwater reaches, allowing for divergence through local adaptation. It is such populations that Minckley and DeMarais [21] hypothesized might be recognized as *Gila nigra*, which is morphologically intermediate to *G*. *intermedia* and *G*. *robusta*. Because the present analysis of microsatellite DNA loci did not identify diagnostic markers for each species, it is impossible to specifically test the introgression hypothesis here. Regardless of the reason, the lack of diagnostic molecular characters to date does not inform the status of *G*. *intermedia*, *G*. *nigra*, and *G*. *robusta* relative to their recognition as distinct species. Instead these results highlight the role that local evolution has played in shaping patterns of variation in these taxa and the importance of accounting for this variation when managing the complex.

### Genetic structure

The high level of genetic subdivision detected in the present study indicates that forces acting on location populations (e.g., mutation, drift, selection) are driving patterns of genetic variation within the *Gila robusta* complex. Similar patterns were identified with allozymes [20] and nuclear and mtDNA sequences [22], indicating that this result does not solely reflect the rapid rate of microsatellite evolution. These patterns more likely reflect varying levels of hydrogeographic connectivity among stream habitats over the last 2–3 million years [2], with samples of *G*. *robusta* (the mainstem species) from the Gila River basin exhibiting increased variability and lower levels of divergence and hierarchical structure than *G*. *nigra* and *G*. *intermedia*, which are typically found in smaller, more isolated streams.

Although the results of the present study support substantial divergence among local populations [20, 22], spatial genetic and clustering analyses performed here indicate that relative impact of evolutionary processes on genetic variation depends on distance between localities, as well as potential barriers to dispersal. For example, *F*-statistic analyses of *G*. *robusta* identified considerable variation in allele frequencies among samples (*F*_ST_ = 0.191); however, removal of samples from the Bill Williams River reduced structure considerably (*F*_ST_ = 0.071). This inference was also supported by scatterplots of pairwise *F*_ST_ versus stream distance (Fig 2B) and a Mantel correlogram (Fig 3B), demonstrating high rates of gene flow relative to drift within the Gila River basin and differentiation between populations from Gila and Bill Williams basins (as well as between sample locations within the Bill Williams basin). While long stream distances separate populations from the Gila and Bill Williams basins, observed differentiation may be best explained by inhospitable habitat in the lowermost Colorado and Gila rivers, which may be acting as an isolating mechanism.

*Gila intermedia* is found in headwater reaches throughout the Gila River drainage and exhibits lower levels of variation within and more differentiation among populations than *G*. *robusta*, as expected. Scatterplots of pairwise *F*_ST_ versus stream distance (Fig 2C) and the Mantel correlogram (Fig 3C) indicate isolation by distance up to some threshold level, beyond which effects of drift predominate [53]. Presence of significant divergence among distant locations likely reflects historical processes attributable to the strongly fluctuating environment [54]. Frequent dry periods would have led to divergence among locations due to drift and selection; gene flow would have been possible during pluvial times. Divergence may have been exacerbated by recent anthropogenic modifications to the stream network that formed barriers to dispersal [55]. While the scatter plot, Mantel correlogram, and clustering analyses indicate that gene flow is more effective than drift at shorter distances (with the effects of drift predominating at longer distances), ongoing hydrogeographic isolation is likely to intensify the strength of drift and erode any signal of localized isolation by distance.

*Gila nigra* also occupies headwater reaches and was also expected to show substantial differentiation among populations, and results based on various types of genetic markers corroborate this inference [20, 22]. While gene flow dynamics generally mirrored those of *G*. *intermedia*, drift induced divergence at long distances was less extreme for *G*. *nigra*, consistent with the observation that genetic variability was uniformly lower for *G*. *intermedia* relative to *G*. *nigra*. There are, however, caveats associated with this interpretation. Sampling of *G*. *nigra* was limited, with half the samples (MAR, SPRSA, ROC, TON) coming from the same relatively small tributary network of the Salt River (Fig 1). Also, the outcome of hierarchical analysis of assignment probabilities indicated that some samples may not be discrete; the samples NMFKS and TURNM were especially problematic, as they may actually be *G*. *robusta* or hybrids (Fig 5).

In general, the results of the spatial genetic analyses indicate that gene flow/drift dynamics depend on stream distance and differ between the mainstem form and the headwater forms. These results support key differences in microevolutionary processes among ecological variants within the *G*. *robusta* complex and should be informative for conservation genetic management of local populations irrespective of species designations.

### Comparison to other fishes from western North America

Results from our study of the *Gila robusta* complex indicate considerable evolution at the local population level but also are consistent with the “Stream Hierarchy” model of gene flow [23], which predicts varying levels of genetic connectivity (and hierarchical structure) within a stream network depending on degree of hydrogeographic connectivity among local populations. Many studies of fishes from desert regions of western North America yield valuable perspective on the role of geographic connectedness and life history on distribution of genetic variation in arid environments. Tibbets and Dowling [26] contrasted levels of divergence among three species of stream-dwelling desert cyprinids (*Agosia chrysogaster*, *Meda fulgida*, and *Tiaroga cobitis*) from the Gila River basin, noting that patterns of genetic variation reflected expectations derived from consideration of life history and predicted levels of movement among locations. Studies of genetic variation in other cyprinids yielded variable results. In their study of mtDNA variation in *Richardsonius*, Houston et al. [56] found most variation distributed among, but not within, major regions, indicating high levels of gene exchange within but not among regions. This contrasts with studies of other minnows (e.g., *Rhinichthys osculus*–[57, 58] and *Lepidomeda*–[59]), where there was considerable divergence among localities within drainages as well as among drainages. Johnson [60] also identified considerable divergence within and among drainage groups in the cyprinid *Gila atraria* but also noted additional divergence associated with evolved life history differences within this species.

Diversity of pattern is not restricted to cyprinids. Whiteley et al. [61] quantified allozyme and microsatellite variation within and among populations of mountain whitefish (*Prosopium williamsoni*) where variation was hierarchically arrayed into five distinct assemblages corresponding to major drainage basins, but with no differentiation within major drainage basins. This contrasts to other salmonids from the same region, which exhibit more divergence among locations than within drainages [62]. Hopken et al. [63] also examined the importance of geographic structure on patterns of genetic variation in bluehead sucker (*Catostomus discobolus*), an endemic to the Colorado River basin. They identified three evolutionarily significant units and seven management units within this species, with each group defined by a geomorphological barrier and/or isolation due to aridity. Together, these studies show that levels of genetic connectivity within drainages can vary among taxa based on hydrogeographical patterns and the life history of a species.

### Conservation implications

Molecular and morphological variation provides critical information for management of this complex. In such situations, it is critical to understand evolutionary processes that generated the underlying genetic diversity, allowing for preservation of the evolutionary legacy and adaptive potential of the complex. To maximize preservation of evolutionary potential, we advocate an approach that preserves available genetic diversity as identified by morphological and molecular analyses. Conservation units should be defined in a hierarchical manner, with genetically distinct units identified within each morphologically recognized species and each subbasin. Importance of local adaptation, drift, and gene flow makes it advisable to consider hydrogeography as well as divergence when developing conservation plans. Note, however, that we found some discrepancies between assignments based on microsatellite data and putative taxonomic status based on morphological traits as defined by Minckley and DeMarais [21]. Because morphological identifications are based upon museum records, such conflicts could represent change in species composition. Given the significance of morphological as well as genetic variation, it is critical that remaining populations of these taxa are characterized to allow for fully informed management of this group.

In addition to maintaining discreteness associated with geographic isolation and evolutionary independence, it is possible that *G*. *nigra* may result from admixture of *G*. *robusta* and *G*. *intermedia*. Connectedness among populations is difficult to envision in today’s environment that includes both physical and biological barriers to exchange; however, there is no obvious resolution of those issues. Instead, we must overcome the general need of placing specific populations into categories and acknowledge that conservation should focus on preserving processes that generate observed patterns as well as the patterns themselves, thus requiring preservation of the entire complex and not just individual species.

Among members of the *Gila robusta* complex only *G*. *intermedia* is listed under the Endangered Species Act, and it thus is the only one to receive protection. There currently is no accommodation for integrated conservation of the complex and little likelihood this will change. Given this restriction, we advocate managing each species independently by sub-drainage, with efforts to avoid mixing stocks from different sub-basins to avert negative consequences associated with outbreeding depression. This requires genetic characterization to match donor and recipient populations prior to translocation. Because of high levels of local differentiation, augmentation should only occur under extreme circumstances (i.e., population collapse, physical evidence of inbreeding depression), with special care to preserve local stocks. Efforts to establish new populations should utilize the nearest geographic population as a source, while avoiding transfer across different subdrainages; this is especially important for headwater forms.

## Supporting Information

S1 Table Genotypes for each locus and individual examined in this study of the *Gila robusta* complex, Arizona—New Mexico. (DOCX)

S2 Table Allelic richness (A_R_) for each locus and sample, including averages, for each sample of the *Gila robusta* complex, Arizona—New Mexico. (DOCX)

S3 Table Gene diversity (unbiased estimate [32]) for each locus and sample of the *Gila robusta* complex, Arizona—New Mexico. (DOCX)
