# Supplementary material for: Population Structure in the Roundtail Chub (Gila robusta Complex) of the Gila River Basin as Determined by Microsatellites: Evolutionary and Conservation Implications
Source: PLoS One. 2015 Oct 16;10(10):e0139832. doi: 10.1371/journal.pone.0139832 (PMC4608781; doi:10.1371/journal.pone.0139832)
Supplement: S1 Table — (DOCX) [file pone.0139832.s001.docx]

**S1 Table. Genotypes for each locus and individual examined in this study of the *Gila robusta* complex, Arizona – New Mexico.** The first column provides the sample identification (acronym provided in Table 1 and specimen number) and following columns provide genotypes for each locus. “0” identifies missing data.
