# Supplementary material for: Population Structure in the Roundtail Chub (Gila robusta Complex) of the Gila River Basin as Determined by Microsatellites: Evolutionary and Conservation Implications
Source: PLoS One. 2015 Oct 16;10(10):e0139832. doi: 10.1371/journal.pone.0139832 (PMC4608781; doi:10.1371/journal.pone.0139832)
Supplement: S2 Table — (DOCX) [file pone.0139832.s002.docx]

**S2 Table. Allelic richness (A_R_) for each locus and sample, including averages, for each sample of the *Gila robusta* complex, Arizona – New Mexico.**
